# Application of biosensors in research of traditional Chinese medicine: a systematic review

**DOI:** 10.1186/s13020-026-01417-w

**Published:** 2026-06-08

**Authors:** Shengnan Du, Fan Zhang, Xing Yan, Liping Huang, Zhixin Wang

**Affiliations:** 1 Jiangxi Province Key Laboratory of Traditional Chinese Medicine Pharmacology, Institute of Traditional Chinese Medicine Health Industry, China Academy of Chinese Medical Sciences, Nanchang, 330115 China; 2 Jiangxi Health Industry Institute of Traditional Chinese Medicine, Nanchang, 330115 China; 3 School of Pharmacy, Jiangxi University of Chinese Medicine, Nanchang, 330004 China

**Keywords:** Biosensor, TCM, Active component, Target of action, Interdisciplinary collaboration

## Abstract

As advanced analytical tools, biosensors combine biological recognition elements with physicochemical transducers, enabling the sensitive and selective detection of target analytes. As research in traditional Chinese medicine (TCM) continues to deepen and shift toward holistic and functional evaluation, biosensors offer significant promise due to their high sensitivity, strong specificity, high throughput, and rapid response. They are expected to serve as an effective bridge linking the chemical constituents of TCM with their pharmacological activities. This review systematically searched the databases including Web of Science, PubMed, Google Scholar and China National Knowledge Infrastructure (CNKI) for literature published from 2001 to 2025. The principles, classifications, advantages, and diverse applications of biosensors in TCM research are summarized, encompassing areas such as herb variety identification, active component analysis and screening, target exploration, drug–target affinity determination, quality control, and safety evaluation. Recent technological advances are highlighted, including innovations in development of novel biosensors, microtechnology and integration, and intelligent data processing. Current limitations and potential countermeasures for overcoming them are also discussed. Finally, the prospects and future research directions of biosensors in TCM research are proposed, emphasizing its potential to drive innovation and accelerate the modernization of TCM industry.

**Graphical Abstract:**

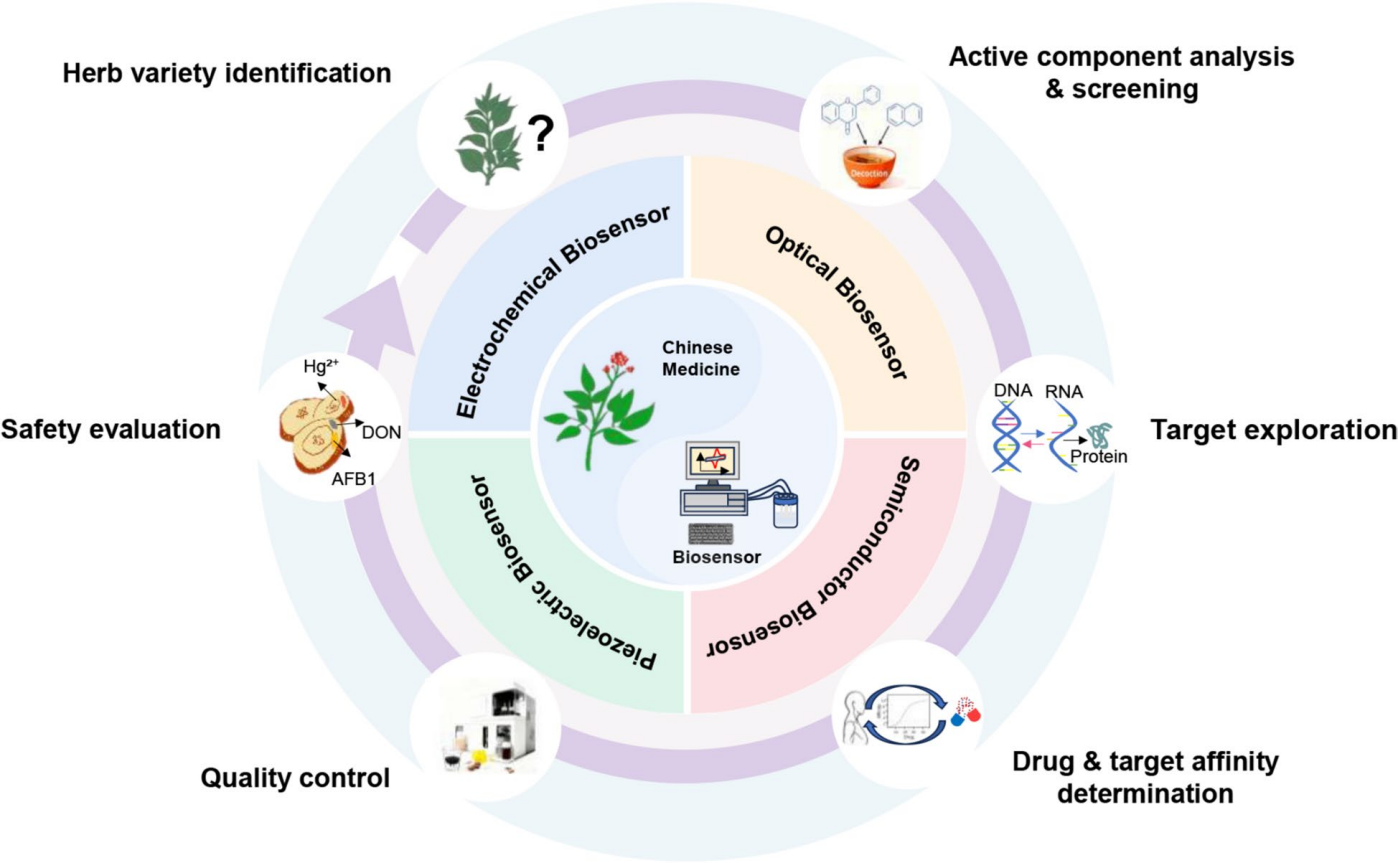

## Introduction

As a time-honored medical system, traditional Chinese medicine (TCM) plays a crucial role in disease prevention, treatment, and health maintenance. It operates under a holistic philosophy that emphasizes the synergistic effects of multiple herbal components [1]. However, this inherent complexity, arising from multi-component formulations, multi-target interactions, and in vivo metabolic transformations, has long posed significant challenges for scientific elucidation and modernization. The lack of effective analytical strategies that can simultaneously capture the chemical diversity, pharmacological relevance, and biological dynamics of TCM remains a major bottleneck for advancing its research and clinical translation.

With the continuous advancement of modern analytical instrumentation, techniques such as liquid chromatography–mass spectrometry (LC–MS) [2], nuclear magnetic resonance spectroscopy [3], and capillary electrophoresis [4] have been widely applied to characterize the chemical composition and metabolic behavior of herbal medicines. These technologies have substantially enhanced the sensitivity, precision, and throughput of TCM analysis, enabling the discovery of numerous bioactive compounds and metabolic markers. Nevertheless, these methods are inherently limited to the qualitative and quantitative characterization of chemical constituents and cannot directly determine whether the detected compounds exhibit biological activity or interact with specific molecular targets. In contrast, biosensors integrate biological recognition elements, including enzymes, receptors, antibodies, and cells, etc., with physicochemical transducers to convert molecular recognition events into measurable signals. Such systems enable the direct evaluation of functional parameters, including enzyme activity, receptor binding affinity, and cellular responses, thereby allowing for highly sensitive, specific, and rapid detection of target analyte [5]. Benefiting from advancements in nanotechnology, microfabrication, and material science, biosensors have demonstrated tremendous potential in a variety of biomedical and pharmaceutical applications. In recent years, their introduction into TCM research has opened new avenues for investigating complex herbal systems. Biosensors have been successfully applied in multiple aspects of TCM research, including the identification of herb varieties [6], analysis and screening of active components [7], exploration of action targets [8], determination of affinity between drug and target [9], quality control [10], and safety monitoring of toxic components or contaminants [11]. These applications highlight the unique ability of biosensors to directly link molecular information with biological function, effectively complementing traditional analytical approaches.

The advancement of biosensors is jointly propelled by the reciprocal promotion between research trends and technological developments [12]. Innovations in nanomaterial-based signal amplification, microfluidic chip integration, and organ-on-chip modeling have significantly improved detection efficiency and reproducibility, while coupling with chromatographic [13] and spectroscopic [14] systems has enhanced analytical accuracy and multi-parameter detection capability. Moreover, the incorporation of machine learning (ML) into biosensor data processing enables intelligent pattern recognition, data fusion, and predictive modeling, paving the way for automated, high-throughput TCM analysis [15]. These integrated technologies collectively contribute to a new paradigm in TCM research, one that emphasizes multidimensional, real-time, and functionally relevant evaluation. Despite these advances, several challenges remain to be addressed before biosensors can be widely implemented in TCM studies. Issues such as the long-term stability and reproducibility of biological recognition elements, interference from complex herbal matrices, limitations in multiplexed analysis, and the lack of standardized methodologies continue to hinder their broader application. Nonetheless, ongoing interdisciplinary efforts in biosensor engineering, synthetic biology, and computational data analysis are expected to overcome these barriers [16].

In summary, biosensors represent a promising technological bridge that connects the chemical complexity and biological essence of TCM. Their continued evolution toward miniaturization, portability, automation, and intelligence will not only enhance analytical precision but also promote the modernization, standardization, and internationalization of TCM. With these developments, biosensors are poised to become indispensable tools for elucidating the material basis and therapeutic mechanisms of TCM in the era of intelligent healthcare.

## Review methodology

This review provides a comprehensive overview of the fundamental principles, classifications, and advantages of biosensors, as well as their specific applications in TCM research. In this context, “TCM research” is defined as non-clinical investigations focusing on TCM materials, decoction pieces, extracts, dispensing granules, and patented medicines. These studies encompass herbal origin identification, material basis, quality control, pharmacological mechanisms, safety evaluation, clinical efficacy, and manufacturing processes. All subsequent discussions are strictly confined to this scope. To ensure a systematic and rigorous analysis, more than 240 representative studies were retrieved from relevant databases, carefully reviewed, and appropriately cited. On this basis, current technological limitations, potential countermeasures, and future development prospects are discussed.Article structure: Kindly check whether the section headings have been identified correctly and amend if any.The manuscript has been revised. Thank you for the reminder.

### Research questions

This review aims to address the following key research questions (RQs):

RQ1: What are the fundamental principles, advantages, and classifications of biosensors employed in TCM research?

RQ2: In which specific areas have biosensors been applied within the context of TCM research?

### Search strategy

Relevant literature was retrieved from major academic databases, including Web of Science, PubMed, Google Scholar, and CNKI. The search was conducted using keywords such as “biosensor”, “Chinese medicine” OR “TCM” OR “herb” with the scope covering article titles, abstracts, and keywords. The retrieved literature was subjected to bibliometric analysis using CiteSpace (https://citespace.podia.com/), which generated a keyword co-occurrence map. After rigorous screening, 246 publications were included in the final analysis. This corpus, spanning 2001 to 2025 and comprising contributions from 32 countries and regions, effectively outlines the research progress in biosensor applications within the field of TCM.

**Fig. 1:**
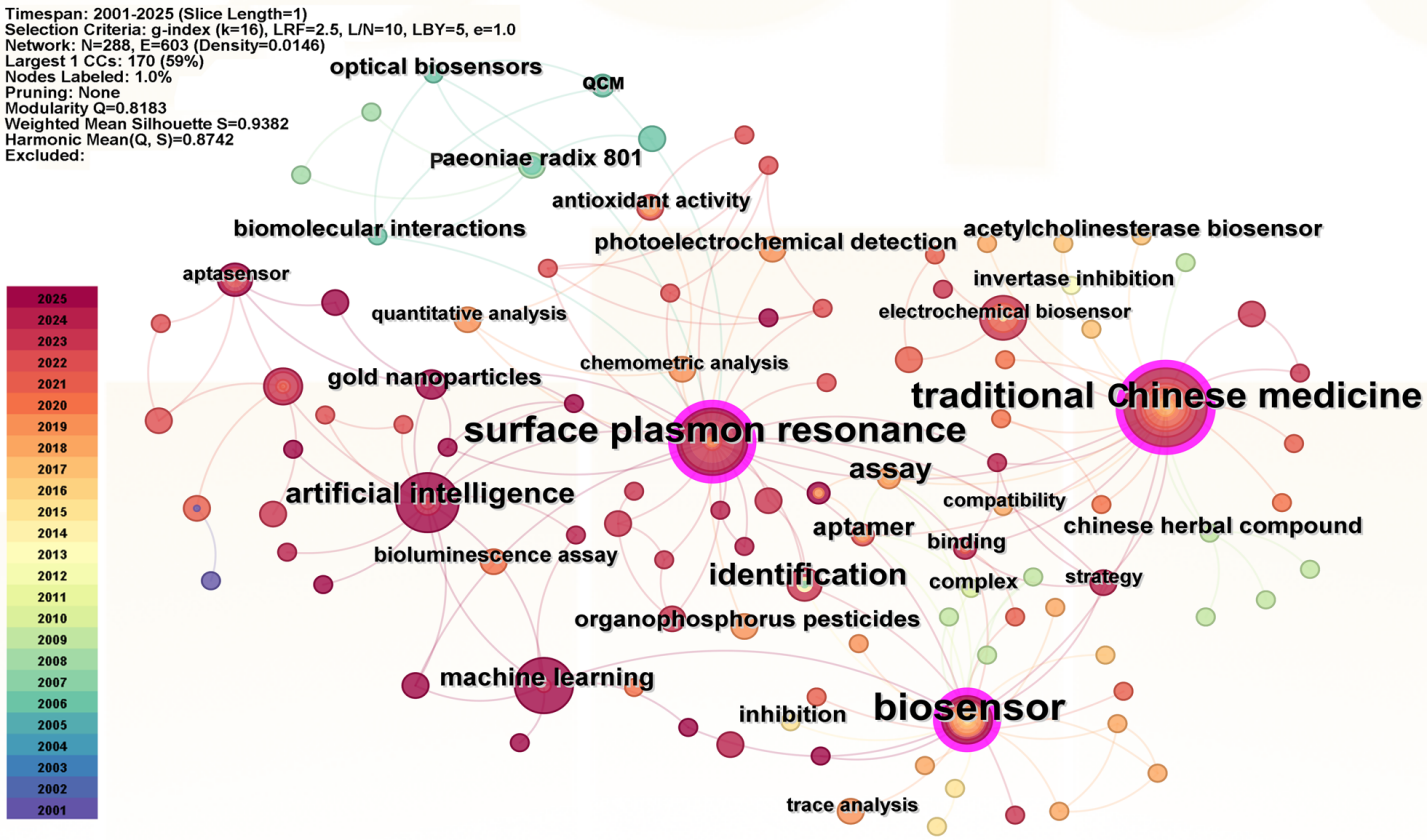
A bibliometric analysis of literature cited in this review performed using CiteSpace. Each node represents a keyword, with its size proportional to frequency of occurrence. Node colors correspond to publication years, where a gradient from cool to warm tones indicates a chronological progression from early to later. Links between nodes denote co-citation relationships, and their thicknesses represent strength of association

A bibliometric analysis based on keywords was performed, and the results are presented in Fig. 1. The CiteSpace analysis employed key parameters to optimize network interpretation: a g-index (k = 16) for scale determination, LRF = 2.5 to balance label clarity, and L/N = 10 to reflect high connectivity. With LBY = 5 and Pathfinder pruning threshold (e = 1.0), the network captured stable thematic trends and significant associations. The resulting network comprised 288 nodes (N = 288) and 603 edges (E = 603), the sparse network (Density = 0.0146) demonstrated broad coverage, where the largest connected component (170 nodes, 59% of the total) revealed central research themes. Strong clustering (Modularity Q = 0.8138; Silhouette S = 0.9382; harmonic mean = 0.8742) validated structural robustness. Analysis of the co-occurrence network identified “TCM” and “surface plasmon resonance (SPR)” as dominant nodes, reflecting the core theme of this study. Furthermore, the co-occurrence of key nodes such as “identification”, “antioxidant activity”, “binding” and “quantitative analysis” reveals diverse applications of biosensors. It is noteworthy that the nodes for “AI” and “ML” exhibited significant growth between 2021 and 2025, indicating that they have emerged as a new research frontier.

### Exclusion screening criteria

To ensure quality and relevance, studies were excluded according to the following criteria:

1. Publication time frame limited to January 2001 through December 2025.
2. Studies lacking detailed information on biosensor types, detection principles, target analytes, sensitivity, specificity, or detection limits were excluded to allow for meaningful cross-comparison.
3. Studies not directly addressing the application of biosensors in TCM research were excluded.

## Principle, classification, and advantages of biosensors

### Fundamental principle of biosensors

**Fig. 2:**
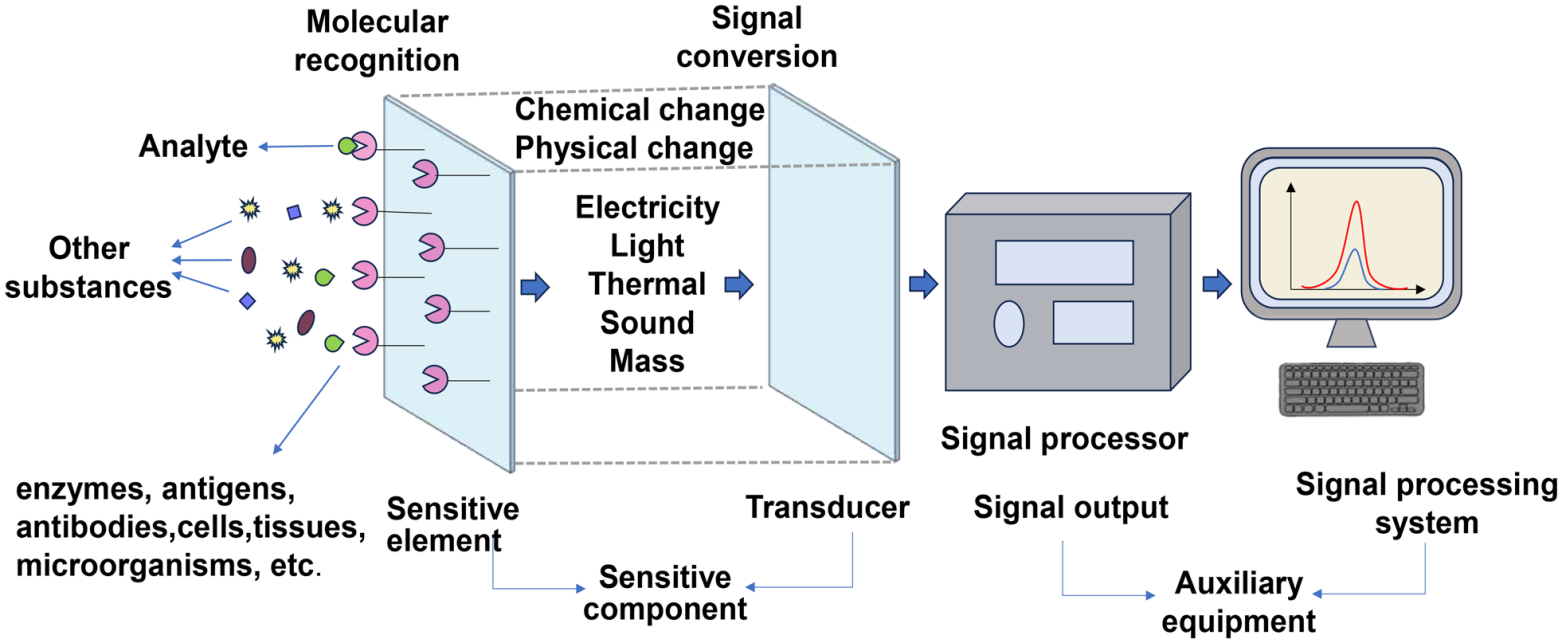
Schematic diagram of components of a biosensor

Biosensors are analytical devices capable of converting biological responses into measurable physical signals [17]. Their working principle involves integrating a biological recognition element with a physicochemical transducer, where the transducer serves as a detector to convert interactions between biological entities and target analytes into detectable signals (Fig. 2) [5]. Fundamentally, a biosensor consists of three essential components. The first component is the biorecognition element, such as enzymes, receptors, antibodies, nucleic acids, cells, microorganisms, tissues, or biomimetic molecules, which possesses molecular recognition capabilities. By specifically binding to or reacting with target molecules, this element induces detectable changes in properties such as light, heat, pH, or mass [18]. The second component is the signal transducer, which may include electrochemical electrodes, SPR systems, field-effect transistors (FETs), or piezoelectric quartz crystals. This component converts the primary signal generated by the biorecognition element into measurable energy outputs, such as electrical, optical, or mechanical signals. The third component is the signal processing system, responsible for amplifying, filtering, and digitally processing the transduced signals. It transforms the outputs into readable numerical or graphical data, thereby enabling accurate analysis and detection of target analytes [19].

### Classification of biosensors

Biosensors, as multi-component devices that integrate biological recognition elements with physicochemical transducers [20], exhibit considerable diversity, giving rise to multiple classification approaches. At present, there is no universally accepted classification system, either domestically or internationally. Among the existing methods, classification based on the type of transducer is the most commonly used, as it provides a clearer framework for understanding biosensor principles and for selecting appropriate devices for specific applications. According to this approach, biosensors can be categorized into electrochemical biosensors, semiconductor biosensors, SPR biosensors, fluorescence biosensors, and others. In this review, biosensors are classified following this transducer-based method. Table 1 summarizes the biosensors that have been successfully applied in the analysis of TCM under this classification scheme.

**Table 1:** Transducer types of biosensors currently applied in TCM research

Transducer type	Principle	Advantages	Disadvantages	Applications	References
Electrochemical	Specific binding of target to the biorecognition element induces a change in the electrochemical properties (e.g. current, potential or resistance) of the electrode surface	a) High sensitivity, low detection limit; b) High selectivity, good specificity; c) Fast response, real-time detection; d) Lower cost, easy to miniaturized and portable; e) Integrable, suitable for on-site detection	a) Susceptible to environmental disturbances; b) Biometric elements are less stable and prone to inactivation; c) Electrode surfaces are prone to contamination; d) Complexity of the preparation process; c) Signals may be interfered with by non-specific adsorptions	Identification of species; analysis and screening of active components; quality control; detection of pesticide residues	[10, 26–28]
Surface plasmon resonance (SPR)	Real-time detection of refractive index changes induced by the binding of biomolecules using the phenomenon of plasma resonance on the surface of metal films	a) Real-time, label-free detection; b) High sensitivity, low detection limit; c) High specificity, good selectivity; d) Monitoring of molecular binding kinetics	a) Higher instrument cost; b) Sensitivity to temperature and vibration; c) Transparency of sample requirements; d) Complexity of data handling	Analysis and screening of active components; exploration of targets; quality control; detection of pesticide residues	[29–32]
Fluorescent	The change in fluorescent signal (intensity, wavelength, or lifetime) following the binding of a fluorescent probe to a target molecule is utilized	a) High sensitivity, low detection limit; b) Strong selectivity, good specificity; c) Fast response time, real-time detection; d) Multi-channel, high-throughput detection; e) Easy to operate, suitable for on-site detection	a) Susceptible to photobleaching and interference with background fluorescence; b) Limited stability of fluorescent probes; c) High sensitivity to environmental factors; d) Matrix-induced interference with fluorescence signals; e) Potential effects of fluorescent labeling on molecular activity	Detection of pesticide residues, heavy metals and pathogenic contamination	[33–35]
Grating	The interaction between biomolecules and the surface of the grating causes changes in the optical parameters of the grating (such as wavelength and intensity), thereby enabling the detection of biomolecules	a) No labelling required, real-time detection; b) High sensitivity, fast response c) Simple structure and ease of miniaturization; d)Can be integrated into microfluidic chips	a) Grating preparation requires high accuracy; b) Sensitive to ambient; temperature and humidity c) Susceptible to non-specific adsorption interference; d) Signal resolution and data processing is more complex	Analysis and screening of active components	[36, 37]
Terahertz	Using the resonance interaction of terahertz waves with subwavelength structures such as metamaterials, by detecting changes in resonance frequency or transmission properties caused by the binding of biomolecules	a) Label-free, non-ionizing, non-destructive; b) It has the ability to “fingerprint” biomolecules; c) It allows for high sensitivity detection	a) Susceptible to environmental humidity interference; b) Detection sensitivity is limited by material properties; c) Equipment cost is high and operation is complicated; d) Signal processing and data analysis is more complicated	Analysis and screening of active components	[38]
Raman spectroscopy	Optical sensing technology based on Raman scattering effect for detection and identification of biomolecules	a) No labelling required; b) High specificity; c) High sensitivity for single molecule detection; d) Suitable for complex samples with high immunity to interference; e) Can be used for in-situ, real-time assays	a) Raman signal is weak and requires enhancement techniques; b) Enhanced substrate preparation is complex; c) Signal is susceptible to interference from fluorescent background; d) Equipment cost is high; e) Data analysis is complex	Quality control	[39]
Immunoaffinity analysis system (IAsys)	Waveguide-coupled laser-generated evanescent fields enable real-time monitoring of molecular interactions by detecting ligand-analyte binding-induced shifts in resonance angle	a) Real-time, label-free detection; b) High sensitivity; c) Kinetic parameters obtained in a single measurement; d) Compatible with a wide range of sample matrices for a wide range of applications	a) Higher cost; b) Sensitive to temperature and vibration; c) Complicated data analysis; d) Relatively limited throughput, difficult to achieve large-scale parallel screening	Analysis and screening of active components	[40]
Optically addressable	Excitation of photogenerated carriers in semiconductor structures using modulated light beams to generate photocurrents, and biorecognition reactions to induce changes in interfacial potentials	a) High spatial resolution for parallel detection of multiple points on a chip; b) No labelling required; c) High sensitivity; d) Suitable for micro-analysis; e) Scalable in combination with microfluidics and other technologies	a) Complex system requiring precision light source and scanning control; b) Sensitive to ambient light, temperature and vibration; c) Complex chip preparation and surface functionalization processes; d) Signal drift is high, requiring frequent calibration; e) Relatively complex data analysis and imaging processing	Analysis and screening of active components	[41]
High electron mobility electron rate tubes (HEMT)	Analyzed by detecting changes in semiconductor surface charge or conductivity caused by the specific binding of a biometric molecule to a target	a) High sensitivity, low detection limit; b) Fast response, real-time detection; c) Easy to miniaturize and integrate; d) Suitable for a wide range of biomolecule detection; e) Enables label-free detection	a) Susceptible to environmental interference; b) Biometric elements are less stable and prone to inactivation; c) Complex and costly preparation process; d) Selectivity needs to be improved and non-specific adsorption may be present; e) Long-term stability and reproducibility still need improvement	Exploration of targets	[42]
Quartz Crystal Microbalance	The quartz crystal oscillates at a specific frequency under an alternating voltage. The surface biometric element captures the target molecule and the mass increases, the oscillation frequency decreases, and the change in frequency is detected to obtain the binding of the target analyte	a) Real-time, label-free detection, preserving molecular activity; b) High sensitivity; c) Fast response, rich in kinetic information; d) Simple structure, easy to miniaturize and array; e) Simple operation, low maintenance costs	a) Sensitive to temperature, pressure and mechanical vibration; b) Significant baseline drift, requiring frequent calibration; c) Difficulty in distinguishing between mass changes and non-mass effects (e.g. viscosity); d) Poor selectivity in complex samples	Exploration of targets	[43]

### Advantages of biosensors

Biosensors offer significant advantages that make them particularly suitable for the analysis of complex systems such as TCM. Unlike conventional analytical tools that primarily focus on the qualitative and quantitative determination of chemical constituents, biosensors integrate biological recognition with physicochemical signal transduction, enabling a direct connection between chemical information and biological function [21]. This feature provides a powerful means to investigate the contributions of active constituents to the pharmacological effects of TCM formulations.

One of the key merits of biosensors lies in their exceptional selectivity and ability to resist interference from complex matrices. By employing highly specific biological recognition elements such as enzymes, antibodies, receptors, aptamers, or molecularly imprinted polymers, biosensors can recognize target analytes even when they are present at trace levels within complex herbal extracts [22]. This inherent specificity allows the detection of active compounds against a background of structurally similar or coexisting substances, which is often challenging for conventional chromatographic or spectroscopic methods. Consequently, biosensors enable more accurate profiling of bioactive molecules and facilitate the identification of compounds directly responsible for pharmacological effects.

In addition to chemical recognition, biosensors provide functional information by translating biological interactions into measurable electrical, optical, or mechanical signals. This capacity allows real-time monitoring of enzymatic reactions, receptor binding events, and cellular responses induced by herbal compounds, thereby revealing the biological activities associated with chemical constituents [23, 24]. Through this integration of detection and activity assessment, biosensors serve as an effective bridge between chemical composition and pharmacodynamics, offering insights into the molecular mechanisms underlying TCM efficacy.

Furthermore, biosensor-based analysis features several operational advantages, including label-free detection, rapid response, and minimal sample preparation [7]. The compatibility with small sample volumes and mild measurement conditions preserves thermolabile compounds and enhances analytical throughput. With ongoing advances in nanomaterials, microfluidics, and signal processing, biosensors have evolved toward miniaturized and portable formats, allowing simultaneous detection of multiple analytes and real-time monitoring in situ [25]. These developments are particularly consistent with the holistic and multitarget nature of TCM, enabling high-efficiency screening and functional evaluation in complex herbal systems.

Overall, biosensors provide an innovative analytical platform that not only characterizes the chemical composition of TCM but also links these constituents with their biological and pharmacological actions. The capacity to achieve selective, rapid, and functionally relevant detection makes biosensors a valuable tool for modernizing TCM research, bridging traditional empirical knowledge with mechanistic understanding and quantitative evaluation.

## Application of biosensors in TCM research

### Identification of herb varieties

Variety authentication serves as the fundamental prerequisite for ensuring the quality, safety, and efficacy of TCM [44]. The primary objective of authenticity assessment is to ensure the accurate identification of medicinal plant species and their specific anatomical parts, thereby preventing adulteration and maintaining clinical consistency. Conventional identification methods, including those based on geographic origin, macroscopic and microscopic examination, and physicochemical profiling, are largely experience-driven and rely heavily on expert judgment. However, these approaches often suffer from substantial subjectivity, a lack of standardized evaluation criteria, and limited capability for comprehensive quality assessment, thereby restricting their applicability in large-scale production and modern quality management systems [45, 46].

Given the inherent limitations of traditional identification techniques, there is an increasing need for advanced analytical technologies to broaden the scope of medicinal plant authentication and improve its reliability. In this context, electrochemical biosensors have emerged as powerful alternative tools for the precise identification of medicinal plant species owing to their high sensitivity, rapid response, good reproducibility, and portability [47].

Lei et al. [48] designed a specific DNA probe targeting a 16-mer sequence located in the 5S rRNA spacer region of *Fritillaria thunbergii* and constructed an electrochemical DNA biosensor for species identification. Because the target sequence is present exclusively in the genome of *F. thunbergii*, the sensor can effectively distinguish this species from *F. cirrhosa* at the DNA level. The sensor exhibited a detection range of 100 fM–10 nM with a detection limit of 11.7 fM and successfully detected PCR-amplified products derived from both *Fritillaria* species. A similar strategy has also been applied to the authentication of saffron (*Crocus sativus*). Shi et al. [49] developed a DNA probe–based electrochemical biosensor capable of specifically hybridizing with the ITS2 DNA barcode of saffron and distinguishing long DNA fragments from closely related adulterant plants, such as *Chrysanthemum indicum* and *Carthamus tinctorius*. The sensor demonstrated sub-femtomolar sensitivity (0.18 fM), single-base resolution, and good reproducibility (RSD = 2.3%). In addition, it effectively minimized the impact of secondary structures in long DNA strands on hybridization efficiency, offering a reliable approach for the rapid on-site identification of TCM. Similar approaches have also been successfully applied to the varietal identification of *Pueraria montana* var. lobata [27]. With continued advances in this field, the application of electrochemical biosensors has expanded from species-level identification to broader taxonomic classification. Wang et al. [6] employed an electrochemical biosensor to obtain electrochemical fingerprints of multiple genera within the Lamiaceae family. Using this approach, 31 species from 22 genera, along with five outgroup samples, were successfully distinguished and clustered into corresponding phylogenetic branches, demonstrating the potential of electrochemical fingerprinting for identifying complex groups of closely related plant taxa.

Collectively, these studies suggest that electrochemical biosensors can serve as a useful analytical platform for identifying medicinal plant species in TCM. Their high sensitivity, rapid response, cost-effectiveness, and ability to capture both chemical and genetic information offer certain advantages over conventional experience-based identification methods. As interface engineering, signal amplification strategies, and chemometric analysis continue to develop, biosensors may become more flexible tools for the accurate identification of Chinese medicinal materials. However, further validation with larger sample sets and standardized protocols is still needed before they can be applied routinely.

## Analysis and screening of active components in TCM

The analysis of bioactive components in TCM is fundamental to elucidating its material basis of efficacy, ensuring quality control, and facilitating the discovery and development of drugs derived from TCM [50]. However, the inherent chemical complexity of TCM, characterized by a multitude of constituents acting synergistically through diverse pathways, poses significant challenges for identifying and characterizing its pharmacologically active components [51–53]. The lack of direct methods to correlate chemical entities with biological activity further complicates the systematic discovery of bioactive molecules.

**Fig. 3:**
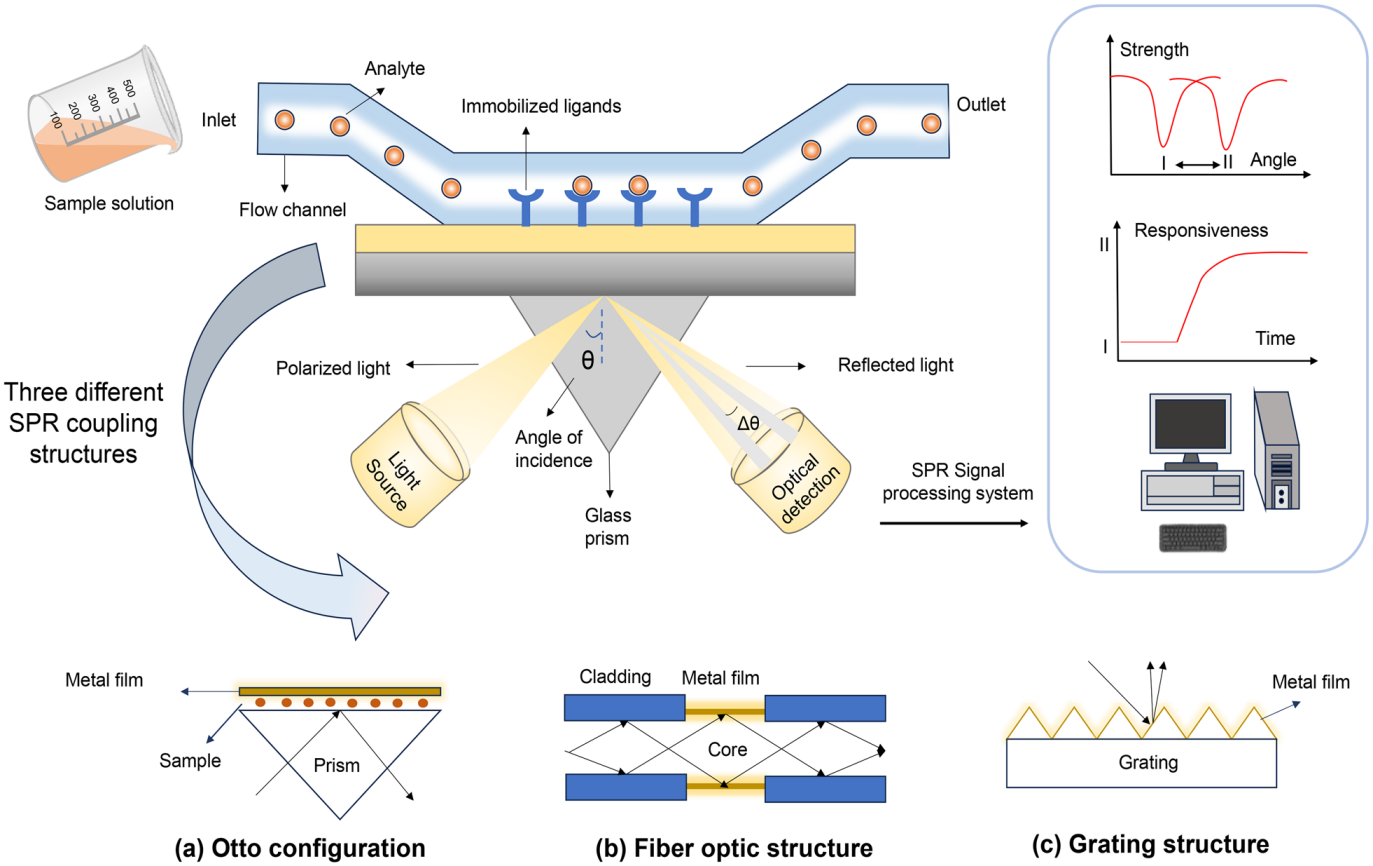
Schematic representation of structure and working principle of an SPR biosensor

Biosensors have emerged as an innovative solution to these challenges, enabling rapid, accurate, and label-free detection of biomolecular interactions, and thereby facilitating the high-throughput screening and functional evaluation of active ingredients in TCM. In recent years, SPR biosensors have been widely adopted in this field and have evolved into one of the most reliable and mature platforms for component analysis. A typical SPR biosensor configuration is shown in Fig. 3.

Li et al. [31] developed a SPR biosensor using xanthine oxidase (XOD) as the biorecognition element to screen active compounds with uric acid-lowering potential in *Chrysanthemum morifolium* Ramat. XOD is a key enzyme that catalyzes the conversion of purines into uric acid, and its inhibitors are widely used in the treatment of hyperuricemia. By immobilizing XOD on the sensor chip surface, the study identified 14 flavonoid compounds from the extract of *Chrysanthemum morifolium* Ramat. Subsequent SPR-based affinity analysis demonstrated that the equilibrium dissociation constants (K_D_) of these compounds toward XOD were within the range of 10⁻⁷–10⁻⁸ M, with luteolin (Lut) and apigenin exhibiting particularly strong binding affinities. Similarly, Chen et al. [54] immobilized lentiviral particles expressing high and low levels of CXCR4 on different flow cells of a CM5 chip to construct a dual-system SPR platform for the identification of bioactive compounds. Using this approach, the study identified senkyunolide I from *Chuanxiong* and demonstrated that it inhibits cell migration, acting as a potential antagonist of the C-X-C chemokine receptor type 4. This work provides a novel perspective for elucidating the anti-inflammatory mechanisms of TCM. Beyond SPR, alternative biosensor modalities have also shown significant potential in the analysis of bioactive compounds. Jiang et al. [55] employed a biosensor ligand screening approach based on SPR, using carbonic anhydrase II (CA II) as the recognition element to identify active constituents from *Kansui* extracts. Their study revealed ingenane type diterpenes as novel agonists of CA II and provided a mechanistic explanation for the enhanced efficacy of vinegar processed *Kansui*. Luo et al. [56] constructed a SPR-based biosensor by immobilizing four target proteins (NFKB1, TNF-α, MMP-9, and MAPK1) onto CM5 sensor chips to validate the interactions between active components in Gu-Ben-Ke-Chuan decoction and chronic bronchitis-related targets. They successfully verified 39 effective components from 53 major constituents in the decoction and demonstrated that these compounds inhibited the release of tumor necrosis factor-α (TNF-α) and IL-6 in a dose-dependent manner, elucidating the potential anti-inflammatory mechanism of GBKC.

According to current research, biosensors provide valuable insights into the direct relationship between chemical constituents and pharmacological activity in the screening of bioactive compounds in TCM. Nevertheless, their successful application to actual TCM samples remains limited, with most studies still at the proof-of-concept stage. Research has primarily concentrated on fundamental aspects, including the optimization of target protein immobilization and the assessment of binding affinities between individual compounds and their targets. Consequently, a substantial gap remains before comprehensive screening of multi-component, low-abundance bioactive compounds in TCM extracts can be realized. Future efforts should prioritize improvements in detection throughput and resistance to matrix interference to facilitate the translation of this approach into practical applications.

### Exploration of targets of TCM action

To advance the modernization of TCM, accurately identifying the molecular targets of active constituents and elucidating their mechanisms of action have become essential objectives of current research [57–59]. The advent of biosensor technology has provided a powerful and versatile approach for investigating these interactions, offering real-time, label-free, and quantitative insights into binding kinetics and affinities between bioactive compounds and their targets. This technological innovation has greatly enhanced the capacity to bridge chemical composition with pharmacological function, revitalizing mechanistic studies in TCM.

Li et al. [40] employed soluble tumor necrosis factor receptor type I (sTNFR-I) as the biorecognition element and utilized an IAsys affinity biosensor to investigate the effects of propyl gallate, an active compound from TCM, on the interaction between TNF-α and sTNFR-I. In this study, sTNFR-I was immobilized on the sensor surface, and real-time monitoring of TNF-α binding kinetics revealed that propyl gallate enhanced the binding affinity between TNF-α and sTNFR-I in a dose-dependent manner. These results suggest that the TNF-α/sTNFR-I interaction may represent a potential mechanism underlying the pharmacological effects of propyl gallate. Similarly, Zhang et al. [32] investigated the treatment of diabetic nephropathy with Gan Di capsules by immobilizing four target proteins, HNF4A, HMGCR, JAK3, and SIRT1, predicted via network pharmacology, on the surface of a CM5 SPR biosensor chip. Real-time, label-free SPR measurements demonstrated that baicalin and scutellarin specifically bound to these four proteins, with K_D_ values ranging from 0.55–178.7 μM. This study underscores the central role of biosensors in TCM research because predicted target molecules can be translated into functionalized sensor surfaces, enabling direct experimental verification of interactions between TCM components and their molecular targets. Such an approach provides a crucial technical foundation for investigating the mechanisms of multi-target, multi-compound therapeutic combinations in TCM. In another study, Ma et al. [60] constructed a high-electron-mobility transistor (HEMT) biosensor using macrophage migration inhibitory factor (MIF) and interleukin-1β (IL-1β) as biorecognition elements to screen bioactive compounds from the TCM formulation Guominkang for the treatment of allergic rhinitis. Using this platform, three key bioactive compounds, 5-O-methylvisamidin, amygdalin, and cimicifugin, were identified. Their K_D_ values ranging from 4.36–48.34 pM for MIF and 6.12 pM–44.86 nM for IL-1β, with most affinities in the picomolar range. This study highlights the potential of biosensors for the direct validation of pharmacological targets and for screening key quality markers in TCM. Wei et al. [9] employed isoquercetin, a bioactive compound derived from TCM, as the biorecognition element. The compound was covalently immobilized onto the gold film surface of a fiber-optic SPR sensor to construct a functionalized sensing interface for target screening. Using this platform, the association and dissociation processes between isoquercetin and three target proteins, PDPK1, INSR, and PTPN1, predicted by virtual screening, were monitored in real time. The results showed that isoquercetin exhibited markedly higher affinity for PTPN1, with a binding to dissociation rate ratio of 13.21, whereas the corresponding values for PDPK1 and INSR were 1.45 and 1.14, respectively. These findings indicate that PTPN1 is the primary molecular target of isoquercetin in the treatment of insulin resistance.

These studies show that biosensors can provide precise, efficient, and real-time analytical tools for validating molecular targets and investigating the mechanisms of action of TCM. Their high sensitivity and specificity support the analysis of complex interactions within multicomponent systems. These features enable biosensors to contribute to target identification and mechanism studies at the molecular level, offering quantifiable and reliable tools for understanding the pharmacological characteristics of TCM, which involves multiple components and targets.

### Determination of affinity between drug and target

Binding kinetics constitute a fundamental aspect of pharmacodynamic evaluation, as they elucidate drug–target interaction mechanisms and inform predictions of efficacy duration, dosage optimization, and inter-individual variability [61–63]. Investigating binding kinetics is thus indispensable for understanding how the inhibitory activity of a compound in vitro translates into its in vivo pharmacological effects.

Tian et al. [64] constructed an electrochemical biosensor by immobilizing double-stranded DNA on a glassy carbon electrode to investigate the interaction between the anticancer herbal compound berberine and DNA. By determining the binding constants and binding site numbers, the study provided quantitative insights into the anticancer mechanism of berberine and laid an experimental foundation for elucidating its pharmacodynamic kinetics. Du et al. [65] established a SPR-based biosensor by immobilizing the SARS-CoV-2 3CLpro protease onto a carboxyl sensor chip for screening potential 3CLpro inhibitors from active ingredients of TCM. Using this sensor, epigallocatechin-3-gallate (EGCG) was validated to exhibit favorable binding affinity to 3CLpro, with a K_D_ value of 6.17 μM. Enzymatic activity inhibition assays further revealed that EGCG effectively suppressed 3CLpro activity, showing an IC_50_ of 0.874 ± 0.005 μM. These results demonstrate that EGCG is a key component responsible for inhibiting SARS-CoV-2 3CLpro activity in the investigated herbal formula. Tsai et al. [66] developed a SPR biosensor by immobilizing human glutaminyl cyclase (hQC) onto a carboxyl-functionalized sensor chip. The binding affinities of two natural flavonoids, azaleatin and quercetin, were determined, with K_D_ values of 0.4 ± 0.1 nM and 5.3 ± 0.5 nM, respectively. Kinetic analysis indicated that both compounds exhibited rapid association and slow dissociation, suggesting strong and stable interactions with hQC.

Collectively, these studies indicate that biosensors can serve as a useful platform for real-time and label-free analysis of binding kinetics between active components of TCM and their molecular targets. By providing kinetic parameters, biosensors allow the evaluation of compound affinity, stability, and pharmacodynamic potential.

### Quality control of TCM

The quality of TCM remains a pivotal yet challenging issue, serving as the cornerstone for ensuring their efficacy and sustainable development [67–69]. Official pharmacopeial methods (e.g., HPLC, GC, TLC) are well-established but are hindered by their reliance on off-line analysis, which involves tedious sample pretreatment, limited throughput, and prolonged analytical cycles. In contrast, biosensors based on electrochemical, optical, or mass spectrometric signal transduction enable in situ, online, and parallel detection of multiple targets in complex matrices, offering advantages such as rapid response, trace-level sensitivity, and high throughput. These features render them transformative for process control and high-throughput screening. However, compared with pharmacopeial methods, biosensors currently exhibit higher variability and necessitate more rigorous validation regarding matrix effects. Therefore, biosensors are not intended to replace official methods but rather to serve as powerful complementary tools. By enabling real-time quality monitoring and rapid screening, they significantly shorten detection cycles, thereby offering a promising technological pathway for modernizing the quality evaluation of TCM preparations.

In TCM quality control research, biosensors based on receptor–ligand interactions provide novel strategies for identifying key quality attributes associated with pharmacological efficacy. Liu et al. [70] developed a HEMT biosensor incorporating taste receptor proteins T1R2, T1R3 and TRPV1 as biorecognition elements to evaluate the interaction strength between active components in Jianwei Xiaoshi Tablets and their corresponding receptors. By integrating this biosensing platform with LC–MS, the study identified 16 chemical constituents in the tablets and further screened six key quality attributes related to sweetness (e.g., naringin and hesperidin) and seven associated with pungency. This approach combines the specific recognition capability of biosensors with the TCM theoretical framework of “nature and taste,” providing new insights for establishing bioactivity-oriented quality evaluation systems for Chinese herbal medicines. Ma et al. [71] developed a macrophage MIF-based HEMT biosensor, in which MIF, a potential therapeutic target for ischemic stroke, was employed as the biorecognition element. The sensor was applied to evaluate the binding affinities of different components in Tongren Niuhuang Qingxin Wan toward MIF. The results demonstrated that all three separated fractions of the formulation exhibited potential therapeutic effects for stroke, with Fraction A showing particularly strong binding affinity to MIF at a concentration of 8.722 × 10⁻^10^ g/mL. By integrating this biosensing platform with ultra-performance LC–MS, the study identified 19 potential key quality attributes associated with MIF-mediated therapeutic effects, including paeoniflorin and cimicifugin. These findings highlight the potential of biosensor-guided screening for establishing the relationship between bioactivity and chemical composition in TCM quality evaluation. Cui et al. [39] established a surface-enhanced Raman scattering biosensor (SERS) biosensor by depositing Ag nanoparticles onto vanadium-titanium-coated cicada wings via magnetron sputtering. The hierarchical nanostructure of the cicada wing enriched amygdalin molecules within the “hot spot” regions, while the localized SPR effect of the Ag nanoparticles enhanced the Raman signals, enabling trace detection of amygdalin with an LOD of 1 × 10⁻⁶ g/L. Combined with ML algorithms, the classification accuracy for R6G spectra at different concentrations reached 83%. Xu et al. [72] designed an electrochemical biosensor for Lut detection based on the specific catalytic activity of laccase toward ortho-diphenol hydroxyl groups. Laccase was immobilized onto TiO₂ nanoparticles and combined with bovine serum albumin-dispersed multi-walled carbon nanotubes, and the composite was modified onto a glassy carbon electrode using chitosan film formation. When Lut diffused to the electrode surface, laccase catalyzed the oxidation of its phenolic hydroxyl groups, simultaneously transferring electrons to the electrode to generate a current signal, which was positively correlated with Lut concentration. The sensor exhibits a wide linear range and a low LOD, and has been successfully applied to the quantitative analysis of Lut in *Lobelia chinensis* and *Taraxacum mongolicum*. As presented above, the analytical performance of the representative biosensors is summarized in Table 2.

**Table 2:** Representative applications of biosensors for quality control of TCM with corresponding analytical performances

No	Analyte	Sample	Detection method	Biorecognition element	Sensing material	Interferent	Linear range	LOD	Recovery	Repeatability (RSD)	Reference
1	Luteolin	*Scutellaria barbata*;Dandelion	Electrochemistry	Laccase	MWCNTs-BSA; TiO₂-laccase; Chitosan	–	80 mM–1 μM; 1–6 μM	11 mM	–	100 cycles: 1.22%; 5 independent electrodes: 10.30%; 10 consecutive measurements: 4.90%	[72]
2	Baicalin	Scutellaria baicalensis glycoside tablets	Electrochemistry	Catalase	AgNCs; BPQDs; MOF	Na⁺; K⁺; Ca^2^⁺; Mg^2^⁺; Zn^2^⁺; Fe^3^⁺; Cu^2^⁺; Glucose; Glutathione; Cysteine	0.01–500 μg/mL	3 ng/mL	96.0%–104.6%	1.20% –3.80%	[24]
3	Ginsenoside Rg1	Standard samples	SPR	Programmed cell death protein 1; Programmed death-ligand 1	Optical fiber; Gold film	Ginsenoside Re; Ginsenoside Rb1; Ginsenoside Rd	–	0.22 μg/mL	–	–	[73]
4	Hyperoside	Standard samples	SPR	Heat shock protein 90α family A member	Optical fiber; Gold film	Isoquercetin; Bicoumarin	–	0.68 μg/mL	–	–	[74]
5	Luteolin	Honeysuckle; *Perilla*	Electrochemistry	Horseradish peroxidase	MWCNTs-CTAB; AuNPs	Ethanol; Glycine; NaCl; Glucose; Ascorbic acid; Thiamine; Uric acid; Puerarin; Baicalin; Naringenin	1 × 10⁻⁸ –2 × 10⁻^5^ M	0.8 nM	98.3%–103.4%; 102.0%–103.3%	3.12%–4.23%; 1.98%–2.15%	[75]
6	Puerarin; Ginsenoside	Human serum & urine	Fluorescence	Puerarin aptamer; ginsenoside aptamer	MoS₂ QDs; CdTe QDs; Polymer brush	Allantoin; β-sitosterol; Riboflavine; Coumarin; Fructose; Vitamin B12; Vitamin B1; Xylitol	0.1–1 μM; 0.05–0.5 μM	36 nM; 8 nM	80%–95%; 90%–102.5%	1.00%–3.34%; 1.00%–2.00%	[76]
7	Berberine	Kampo medicine formulations	Fluorescence	Aptamer	TetBBR38S	Dihydroberberine; Coptisine	0.780–50.0 μg/mL	0.369 μg/mL	90.8%–112%	Intra-assay: 0.331%–2.27%; inter-assay: 1.30%–4.17%	[77]

Overall, biosensors, owing to their speed, miniaturization, and online detection capability, show broad potential for in-process monitoring (e.g., real-time tracking during extraction and concentration), rapid screening of finished products, and on-site inspection, thereby enhancing efficiency in quality control of TCM preparations. Nevertheless, their quantitative accuracy and long-term stability still require calibration and validation against conventional methods. As a powerful complement to traditional analytical systems, biosensors are evolving synergistically with modern instrumental technologies, jointly advancing TCM quality evaluation toward a more intelligent, real-time, and precise paradigm.

### Safety evaluation of TCM

The harmful substances in TCM can generally be classified into two categories: endogenous and exogenous [78]. Exogenous harmful substances are introduced during the cultivation, processing, storage, and transportation stages rather than being naturally present in the herbs. These contaminants may pose potential health risks and mainly include pesticide residues, heavy metals and other toxic elements, as well as mycotoxins [79–81]. At present, biosensor technology has been primarily applied to detect such exogenous contaminants, serving as an important tool in the safety evaluation of TCM. The representative applications and analytical performance of the aforementioned biosensors for the detection of exogenous harmful substances in TCM, including heavy metals, pesticide residues, and pathogenic microorganisms, are summarized in Table 3.

**Table 3:** Representative applications of biosensors for detection of pesticide residues, heavy metals and pathogenic contaminations in TCM with corresponding analytical performances

Application	Analyte	Sample	Matrix	Detection method	Biorecognition element	Sensing material	Interferant	Linear range	LOD	Recovery	Repeatability (RSD)	Reference
Pesticide residue detection	Glyphosate	Honeysuckle	Solution	Fluorescent	Nucleic acids	(Z)-N'-(5-(4-(diphenylamino)phenyl)thiophen-2-yl)methylene)picolinohydrazide	NO₃⁻; S₂O₃^2^⁻; HSO₃⁻; HCO₃⁻; ClO₄⁻; Cl⁻; HPO₄^2^⁻; BF₄⁻; F⁻; CrO₄^2^⁻; I⁻; Phenylalanine; Alanine; Glutamic acid; Dichlorvos; Parathion; Dimethoate; Methyl parathion; Malathion;	5–16 μM	0.588 μM	103.2%–105.2%	2.12%–2.6%	[33]
	Methyl parathion	*Cistanche deserticola*	Sample dissolved in PBS	Electrochemistry	Acetylcholinesterase	AuNPs/PB/CS@N-Gr	L-methionine; Glycine; Glucose; Deltamethrin; Urea	1 × 10⁻^3^–1 × 10^1^ μg	9.47 × 10⁻^5^ μg	98.6%–102.8%	1.84% –2.64%	[22]
	Chlorpyrifos	*Astragalus*	Sample dissolved in PBS	Electrochemistry	Acetylcholinesterase	PANI/AuNPs	Glycine; Glyphosate; Soluble starch; Urea; Ascorbic acid	1.00 × 10⁻^3^–1.00 × 10^1^ ppm	7.90 × 10⁻^5^ ppm	96.68%–102.32%	1.97%–2.41%	[87]
	Acetamiprid	Yam; *Sophora flavescens*	Sample dissolved in PBS	Fluorescent	Nucleic acids	oxSWCNHs	Fipronil; Carbendazim; Pymetrozine; Ethiprole; Dinotefuran	10–90 ng	6.33 ng	96.2%–109.6% 92.8%–105.8%	0.5% –1.2%	[26]
	Aoumaphos	*Angelica Sinensis*	Solution	Fluorescent	Acetylcholinesterase; ChOx	GQDs@GSH	Leucine; Serine; Tyrosine; Threonine; Histidine; Arginine; Lysine; Phenylalanine; Cholesterol; Glycine; Aspartic acid; Tryptophan; Glucose	0.1–10.0 μm	0.075 μM	< 1.98%	101.44%–117.90%	[34]
	Carbendazim	Medlar	Solution	SPR	Anti-MBC monoclonal antibody; MBC-mAb	Au/Fe₃O₄	Benzimidazole; 2-mercaptobenzimidazole; 2-benzimidazole propionic acid; 2-(2-aminoethyl) benzimidazole	0.05–150 ng/mL	0.44 ng/mL	102.4%–115.0%	7.4%–9.2%	[30]
	Glyphosate; Fluorophosphate; Chlorpyrifos	Polygonatum cyrtonea Hua	Water extract samples of Polygonatum cyrtonea Hua	Fluorescent	Acetylcholinesterase; Choline oxidase	PC–CQDs	Fe^3^⁺; Cu⁺; Cu^2^⁺; NH₄⁺; Zr^4^⁺; Fe^2^⁺; Ca^2^⁺; Co^2^⁺; Mg^2^⁺; K⁺; Al^3^⁺; Zn^2^⁺; IO₄⁻; MoO₄^2^⁻; NO₂⁻; WO₄^2^⁻; CO₃^2^⁻; S₂O₃^2^⁻; F⁻, HCO₃⁻; I⁻; Br⁻; SO₄^2^⁻	4 × 10⁻^3^–2 × 10⁻^2^ ppm	1.74 × 10⁻^3^–3.99 × 10⁻^3^ ppm	92.14%–101.81%	2.77%–12.35%	[99]
Heavy metal residue detection	Pb^2^⁺	*Astragalus membranaceus; Glycyrrhiza uralensis*	Dissolve in the test buffer	Electrochemistry	Peptide	Porous anodized aluminum membrane	As^3^⁺; Cd^2^⁺; Co^2^⁺; Cr^3^⁺; Cu^2^⁺; Fe^3^⁺; Hg^2^⁺; Mg^2^⁺; Mn^2^⁺; Ni^2^⁺; Zn^2^⁺	0.01–100 μM	0.005 μM	87.7% –116.8%	–	[91]
	Hg^2^⁺	*Ophiocordyceps sinensis*	Dissolve in the test buffer	Electrochemistry	Nucleic acids	Tetraferrocene	Ca^2^⁺; Mg^2^⁺; Al^3^⁺; Ba^2^⁺; Cd^2^⁺; Cu^2^⁺; Cr^2^⁺; Ni^2^⁺; Zn^2^⁺; Co^2^⁺	0.2–2000 pM	0.12 pM	94.5%–106.2%	2.18% –10.12%	[78]
	Cu^2^⁺	Honeysuckle	Herbal extract	Fluorescent	Nucleic acids	(Z)-N'-(5-(4-(diphenylamino)phenyl)thiophen-2-yl)methylene)picolinohydrazide	Al^3^⁺; K⁺; Mg^2^⁺; Ni^2^⁺; Fe^3^⁺; Fe^2^⁺; Mn^2^⁺; Cr^3^⁺; Ba^2^⁺; Hg^2^⁺; Pb^2^⁺; Cd^2^⁺; Co^2^⁺; Ca^2^⁺; Na⁺; Zn^2^⁺; Ag⁺	1–8.5 μM	2.5 μM	90.4%–102.3%	2.54%–5.75%	[33]
	Cd^2^⁺	*Panax notoginseng*	Solution	Fluorescent	Nucleic acids	NCDs	Co^2^⁺; Li⁺; Zr^4^⁺; As^3^⁺	0–60 ng	0.89 ng	86.50%–104.09%	0.22%–2.74%	[100]
	Pb^2^⁺	*Bauhinia championi*	Solution	Electrochemistry	Enzymes	RuHex	Ag⁺; Hg^2^⁺; Zn^2^⁺; Cu^2^⁺; Cd^2^⁺; Ni^2^⁺; Fe^3^⁺; Al^3^⁺	0.05 –200 nM	0.02 nM	95.5%–115%	–	[101]
Pathogenic contamination detection	Ochratoxin A	*Astragalus membranaceus*	Solution	Lateral flow aptasensor	Nucleic acids	Magnetic nanoparticles	Ochratoxin B; Ochratoxin C; Warfarin; Aflatoxin B1; Zearalenone; Deoxynivalenol;Fumonisin B1	0.2–20 ng/mL	0.053 ng/mL	Approaching 100%	< 10%	[102]
	Ochratoxin A	*Astragalus membranaceus*	Solution	Photoelectrochemical aptasensor	Nucleic acid	BiOI/Cu₂O@Au	Ochratoxin B; Aflatoxin B1; Zearalenone	10 fg/mL–10 ng/mL	2.5 fg/mL	98.7%–103.0%	< 2.0%	[103]
	Aflatoxin B1	*Rehmannia glutinosa* Yam; Tribute chrysanthemum; Malt	Solution	Fluorescent	Aptamer; Antibody	ARGET-ATRP	Aflatoxin B2; Aflatoxin G1; Aflatoxin G2	100 fg/mL–100 ng/mL	8.38 fg/mL	90.52%–98.45%	0.94%–5.6%	[97]
	Ochratoxin A	Forsythia; Astragalus; Licorice;Xanthium; Malt	Solution	Fluorescent	Aptamer	ARGET ATRP	Ochratoxin; Ochratoxin C; Aflatoxin B1; Zearalenone	2 × 10⁻^3^ –2 × 10^3^ ng/mL	7.6 fg/mL	90.00%–105.00%	1.13%–4.55%	[35]
	Deoxynivalenol	Malt; Peach seed	Solution	Electrochemistry	Aptamer; Nucleic acid	NH₂-MIL-101@MoS₂	Zearalenone; Aflatoxin B1	1 fg/mL–10 ng/mL	0.31 fg/mL	92.4%–108%; 92.5%–100%	0.72%–5.67%	[95]
	Aflatoxin B1	Leech	Solution	Electroluminescence	Anti-aflatoxin B1 antibody	AuNCs@CS@GNPs	Aflatoxin B2; Aflatoxin B2; Zearalenone	3.16 × 10⁻^14^–3.16 × 10⁻^12^ g/mL	9.3 × 10⁻^15^ g/mL	97.9%–104.5%	Intra-day (n = 5): 0.04% inter-day (5 days): 5.2%	[14]
	Ochratoxin A	*Morinda officinalis*	Solution	Electrochemistry; Electrochemiluminescence	Aptamer	CRISPR-Cas12a	Ochratoxin B; Ochratoxin C;	1–5000 pg/mL	0.29 pg/mL; 0.37 pg/mL	96%–107.5% 90.9%–111.8%	3.7%–6.8%	[104]
	Aflatoxin B1	Coix seed; Polygala root	Solution	Electrochemistry	Aptamer	3D-rGO/Au NPs	Aflatoxin B2; Aflatoxin G2; Deoxynivalenol; Fumonisin B1; Ochratoxin A; Zearalenone	0.01–100 pg/mL	2.84 fg/mL	94.0%–104.8%;95%–96%	3.16%–5.63%	[105]
	Aflatoxin B1	Suzi; Baiqian; Qianhu; Sangdian Erythrina; Platycodonopsis; Chenpi; Scutellariae Radix; Sizzling Baibai; Sizzling Licorice; Sizzling Asteraceae; Sizzling Fengyuan; Maitong	Solution	Electrochemical	Nucleic acids	Au/N-CNFs/CFs	Ochratoxin A; Zearalenone; Aflatoxin M1; Aflatoxin B2	10.0 –1.0 × 10⁸ pg/mL	6.4 pg/mL	96.18%–112.87%	1.6%	[106]
	Salmonella typhimurium	Niu Huang Qing Xin Wan	Solution	Microfluidic optical biosensor	Monoclonal antibody; Anti-Salmonella polyclonal antibody	Au@PtNCs@MNBs	–	9 × 10^1^–9 × 10^5^ CFU/mL	90 CFU/mL	76.8%–109.5%	–	[107]
	*Escherichia coli*	Licorice	Solution	Electrochemical	Aptamer	SH-capture probe; Biotinylated aptamer probe	Licorice extract	5 × 10^2^–5 × 10⁷ CFU/mL	80 CFU/mL	–	1.1%–5.3%	[108]

#### Pesticide residue detection

Chinese medicinal materials form the foundation of the TCM industry, and their quality directly influences the sector’s development [82]. In recent years, wild resources have become increasingly scarce, unable to meet market demand [83], making large-scale cultivation essential for ensuring a stable supply [84]. During cultivation, pesticides are widely applied to control pests and diseases, thereby improving yield and quality [85]. However, excessive or improper pesticide use can result in residue accumulation, posing acute and chronic toxic risks to humans, animals, and the environment [86].

*Angelica sinensis* (Chinese angelica), a widely used medicinal and edible plant, is often treated with organophosphorus pesticides (OPPs), making OPP residues a key quality indicator. Mu et al. [34] developed an off–on-off–on fluorescent biosensor based on glutathione-modified graphene quantum dots (GQDs@GSH). This sensor employs a multi-step signal modulation mechanism to convert the inhibitory effect of pesticides on acetylcholinesterase (AChE) into measurable fluorescence changes, thereby making the signal highly dependent on the specific enzymatic reaction and effectively reducing nonspecific matrix interference. The sensor was successfully applied to the determination of phoxim in *Angelica sinensis*, exhibiting a good linear response, satisfactory recovery, and high reproducibility. Sun et al. [22] constructed an electrochemical biosensor for the detection of methyl parathion residues in TCM based on the enzyme inhibition principle of AChE. The detection mechanism relies on the fact that methyl parathion, as a specific inhibitor of AChE, effectively suppresses enzyme activity, leading to reduced production of thiocholine and a consequent decrease in the electrochemical signal response. By establishing a quantitative relationship between the inhibition rate and methyl parathion concentration, indirect quantification of the target analyte is achieved. Under optimized conditions, the sensor demonstrated a good linear response and a low LOD. It was further validated through spiked recovery experiments in *Cistanche deserticola* samples, showing satisfactory recovery and reproducibility. The same team [87] subsequently developed a sensor based on polyaniline and gold nanoparticles for the detection of chlorpyrifos residues, achieving a LOD in *Astragalus membranaceus* samples, along with satisfactory spiked recovery results. Feng et al. [26] developed a fluorescence aptamer-based biosensor for the detection of acetamiprid residues in TCM, employing a nucleic acid aptamer as the recognition unit, in combination with the fluorescence quenching capability of oxidized single-walled carbon nanohorns (oxSWCNHs) and cryonase-assisted signal amplification. In the absence of acetamiprid, the FAM-labelled aptamer is adsorbed onto the surface of oxSWCNHs through π–π stacking interactions, resulting in fluorescence quenching. Upon the introduction of acetamiprid, the aptamer binds specifically to the target to form a complex, which cannot be adsorbed onto the oxSWCNHs surface, thereby restoring fluorescence. Subsequently, cryonase degrades the aptamer, releasing acetamiprid and enabling it to participate in further recognition cycles, thereby achieving cyclic signal amplification. This mechanism ensures that the fluorescence signal originates exclusively from specific target recognition events, effectively reducing interference from non-specific substances. The sensor was evaluated using spiked recovery experiments in TCM samples, including *Dioscorea opposita* and *Sophora flavescens*, and demonstrated satisfactory recovery and high reproducibility. The aptamer exhibits high specificity, with negligible response to the structural analog imidacloprid, highlighting its superior selectivity compared with conventional enzyme inhibition and immunoassay based methods.

Overall, biosensors show good stability, reproducibility, and sensitivity in the detection of pesticide residues in TCM. They enable trace-level detection, support quality assessment, and can serve as a technical basis for the development of portable, on-site detection platforms, providing useful support for regulatory monitoring and related applications.

#### Heavy metal residue detection

In recent years, increasing reports of excessive heavy metal content in TCM have raised widespread concern. Heavy metal contamination not only undermines the quality and safety of medicinal materials, resulting in economic losses and export restrictions, but also damages the international reputation of TCM [59, 88, 89]. Long-term exposure to elevated heavy metal levels also poses serious health risks [90].

Tu et al. [91] developed a nanochannel biosensor for the detection of lead ions in TCM, using a Pb^2+^ specific peptide as the biological recognition element. In the presence of Pb^2+^, the peptide selectively binds to the target ion, inducing conformational folding and increasing the positive charge on the channel surface, which in turn significantly enhances ionic current within the nanochannel. The biosensor exhibits excellent selectivity toward Pb^2+^, showing negligible responses to potential interfering ions such as As^3+^, Cd^2+^, and Co^2+^. Under optimized conditions, the sensor demonstrates a wide linear response range and a low LOD. It has been successfully applied to the determination of Pb^2+^ in real samples, including *Astragalus membranaceus* and *Glycyrrhiza uralensis*, yielding satisfactory recovery rates in comparison with atomic absorption spectrometry. Zhong et al. [92] constructed an electrochemical biosensor for the detection of Hg^2^⁺ in TCM based on T-Hg^2^⁺-T coordination chemistry, using thymine-rich DNA hairpin probes as the recognition element in combination with catalytic hairpin assembly signal amplification and tetraferrocene as the electrochemical label. Upon introduction of Hg^2^⁺, the target triggered the self-assembly of hairpin probes mediated by auxiliary DNA, forming a rigid DNA triangle structure. This structure was rapidly immobilized onto the bare gold electrode surface via potential-assisted Au–S self-assembly, bringing tetraferrocene close to the electrode and generating an electrochemical signal. The dual signal amplification was achieved through catalytic hairpin assembly and the multi-electron transfer property of tetraferrocene. The sensor exhibited a good linear response over the concentration range of 0.2–2 × 10^3^ pM, with an LOD of 0.12 pM. It was successfully applied to the determination of Hg^2^⁺ in *Ophiocordyceps sinensis*, with recoveries ranging from 94.5%–106.2% and RSD below 10.12%. Liu et al. [33] designed a fluorescent biosensor that employs a triphenylamine based Schiff base molecule, MPH, as the biological recognition element. The sensor achieves highly selective detection of Cu^2+^ through specific functional groups within the MPH molecule, including pyridine nitrogen and imine nitrogen atoms. Based on this recognition mechanism, the sensor exhibits good sensitivity and selectivity toward Cu^2+^ and has been successfully applied to the determination of Cu^2+^ in extracts of *Lonicera japonica*.

At present, biosensors show potential for the detection of heavy metals in TCM, with advantages such as simple operation, minimal instrumentation, and reduced sample preparation. Under optimized conditions, they can achieve sensitive and selective detection in certain complex matrices, including those containing interfering ions, suggesting potential applications in quality control. With further refinement and validation using real-world samples, such platforms may be considered for integration into commercial testing systems for monitoring multiple heavy metal residues in TCM.

#### Pathogenic contamination detection

Chinese medicinal materials are vulnerable to mycotoxin contamination throughout cultivation, harvesting, processing, transportation, and storage, especially in materials rich in starch, polysaccharides, or lipids [93, 94]. Mycotoxins such as aflatoxins, ochratoxins, and deoxynivalenol (DON) are secondary metabolites with carcinogenic, teratogenic, and mutagenic effects, posing serious health risks [95]. Aflatoxin B1 (AFB1), among the most toxic aflatoxins, is commonly detected in grains, herbal medicines, nuts, and dried fruits, and has been classified as a human carcinogen by the International Agency for Research on Cancer [96].

Guo et al. [97] developed a fluorescent biosensor based on a signal amplification strategy involving electron transfer and atom transfer radical polymerization. The sensor integrates a long-wavelength carboxylated porphyrin as the signal reporter with carboxyl-modified magnetic beads via a spatial separation design, thereby effectively reducing interference from complex TCM matrices. This biosensor enables highly sensitive detection of AFB1 in a variety of TCM, including yam, *Rehmannia glutinosa*, *Tribute chrysanthemum* and malt. and exhibits high accuracy and reproducibility. Cui et al. [35] developed a signal-off fluorescent biosensor based on activators regenerated by electron transfer atom transfer radical polymerization (ARGET ATRP) signal amplification strategy, using an Ochratoxin A (OTA)-specific nucleic acid aptamer as the biorecognition element. In the presence of OTA, the aptamer preferentially binds to the target, leading to the dissociation of the complementary strand carrying the fluorescent polymer from the surface of the magnetic beads, which results in a significant decrease in fluorescence intensity. This signal-off mode, combined with magnetic separation, effectively minimizes false-positive interference caused by nonspecific adsorption in complex TCM. The sensor was successfully applied to five herbal matrices, including *Forsythia suspensa*, *Astragalus membranaceus*, *Glycyrrhiza uralensis*, *Xanthium sibiricum*, and malt, with recovery rates ranging from 90.00%–105.00% and RSD values below 4.55%, demonstrating strong anti-interference capability and reliable performance in complex matrices. Liu et al. [98] constructed a dual-signal electrochemical aptasensor based on NH₂-MIL-101@MoS₂ nanocomposites. By simplifying sample pretreatment using acetonitrile and water extraction and incorporating a dual signal ratio metric strategy with internal self-calibration, the developed sensor effectively suppressed signal drift caused by organic acids and pigments in TCM matrices. This method enabled highly sensitive detection of deoxynivalenol (DON), achieving a LOD of 0.31 fg/mL and a linear range of 1 fg/mL–10 ng/mL. Spike recoveries ranged from 92.4%–108% in malt and 92.5%–100% in peach seed samples, with RSDs below 5.7%.

Biosensors leverage biological recognition elements, including antibodies and nucleic acid aptamers, to specifically detect individual mycotoxins, providing a reliable platform for monitoring TCM safety. However, the performance of biosensors for heavy metal ions is highly dependent on the structural and functional stability of the biological materials, which are sensitive to environmental conditions. Advances in biotechnology, alongside deeper understanding of biomaterial-metal interactions, can improve biosensor reliability. Future developments should integrate cutting-edge technologies such as nanotechnology and synthetic biology to enhance performance, overcome existing limitations, and realize practical, robust detection instruments for routine TCM safety evaluation.

## Technological advances of biosensors in TCM research

### Development of novel biosensors

**Fig. 4:**
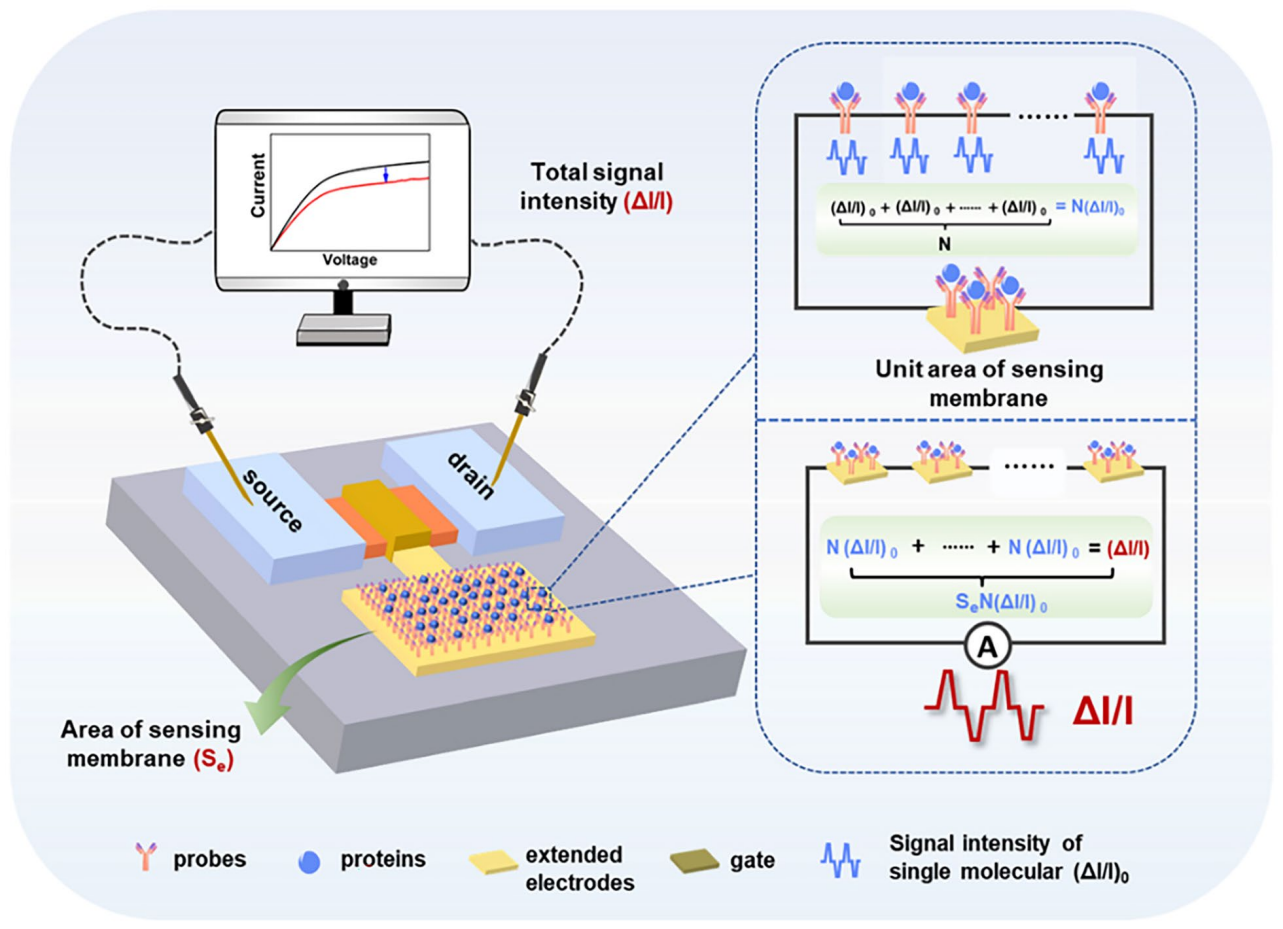
Schematic diagram of basic structure and operating principle of an extended gate HEMT biosensor. Reprinted from Cui et al. [119] with the permission from Institute of Physics (IOP) Publishing

In the field of TCM research, the application of novel biosensors is gradually gaining prominence, such as semiconductor biosensors [109], nano-biosensors, and more [110]. Semiconductor biosensors, such as HEMTs, with their high sensitivity and selectivity, provide advantageous tools for detecting TCM components and exploring their mechanisms of action [111]. Among them, HEMT biosensors have shown significant potential in discovering natural small molecules in TCM that interact strongly with key disease biomarkers [112]. The operation of a typical AlGaN/GaN-based HEMT relies on the unique working mechanism of the two-dimensional electron gas (2DEG). Unlike conventional FETs, in which the conductive channel is formed within a doped silicon layer, the channel in HEMTs is induced by polarization effects at the heterointerface, allowing high electron mobility and high carrier density to be achieved without the need for impurity doping. When target biomolecules are captured by recognition elements immobilized on the gate sensing surface, the resulting change in surface charge density modulates the 2DEG concentration via the field effect, which is subsequently transduced into a measurable drain current response, as illustrated in Fig. 4. For instance, Tang et al. [113] constructed a Syk/Lyn/Fyn-functionalized HEMT biosensor and successfully identified the main anti-allergic active ingredients from the TCM prescription Guomingkang, namely hamaudol and emodin. Ma et al. [42] developed three highly sensitive and highly specific extended gate HEMT biosensors, which have been successfully applied to study the interaction between apoptosis and autophagy. Studies indicate that schisandrin A regulates the autophagy and apoptosis of sperm cells, aiding in repairing damaged testicular tissue to improve sperm quality and alleviate symptoms of oligoasthenozoospermia, with TRPV1 and c-kit considered as potential key targets. Compared to other sensors, this HEMT biosensor, through extending the gate channel, has raised the LOD to 2.787 fg/L, the quantification limit to 0.1 pg/L, expanding the quantification range from 2 to 3 orders of magnitude of the traditional sensors to 5 orders of magnitude (0.1 pg/L–1.0 ng/L). This enhancement significantly improves the detection capability and application potential of biosensors in complex biological systems.

**Fig. 5:**
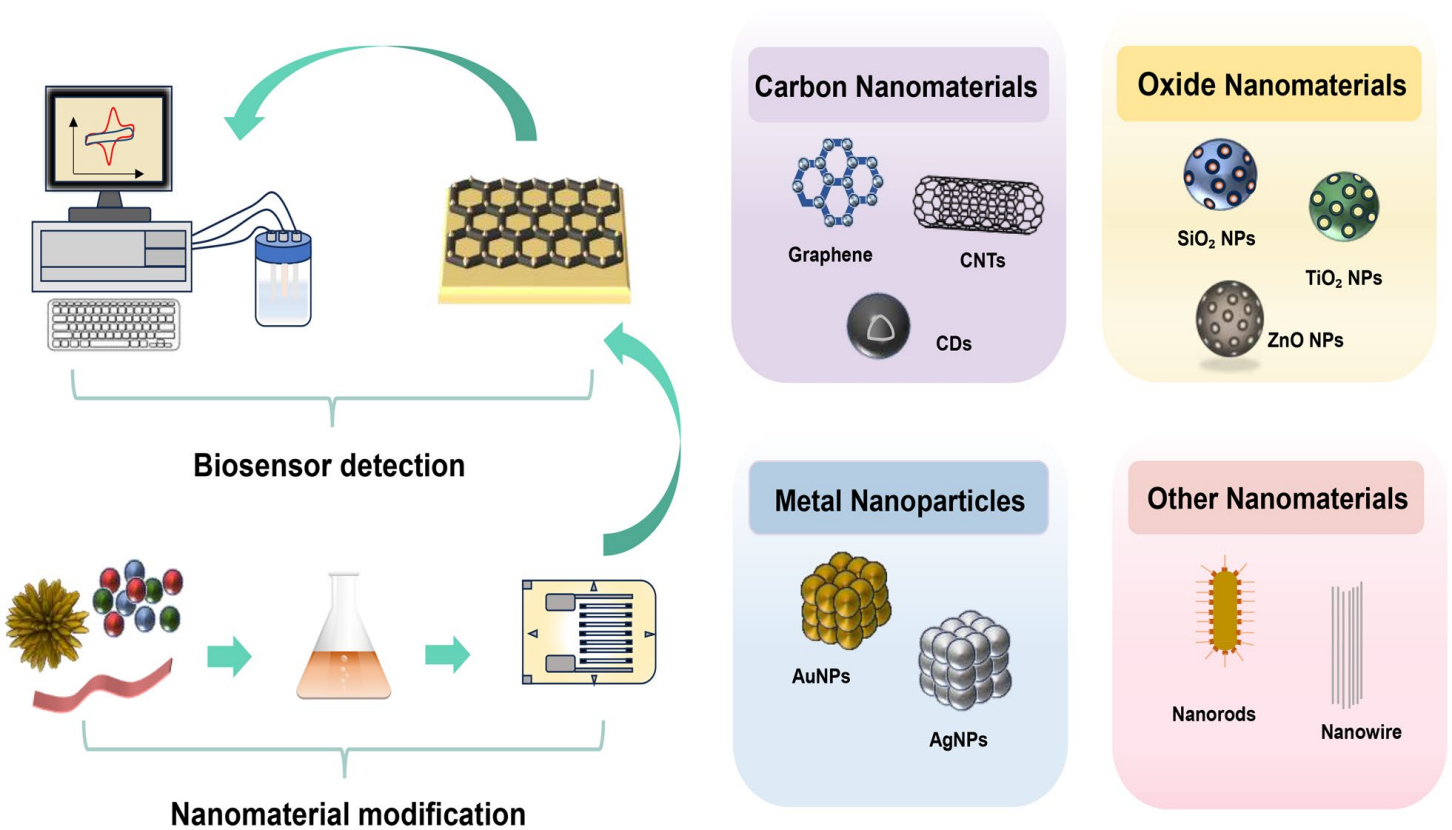
Schematic representation of nanomaterials applied to biosensors. Left: workflow of biosensors constructed using nanomaterials. Right: classification of nanomaterials applied to biosensors with examples

In addition to semiconductor biosensors, entering the twenty-first century, with the rapid development of nanotechnology, nanomaterials have shown significant advantages in multiple fields [114]. For biosensors, the unique properties of nanomaterials in optical performance, electrical performance, chemical activity, etc., make them excellent transducer components [115]. Figure 5 includes nanomaterials commonly used to construct biosensors. On the other hand, the molecular operation of biosensors is essentially based on the nanoscale. The application of nanomaterials can integrate their excellent characteristics (such as high conductivity, stability, biocompatibility, etc.) into the molecular operation system, thereby optimizing or even revolutionizing the operation mechanism of molecules and enhancing the overall performance of sensors [116, 117]. Sun et al. [118] designed an electrochemical biosensor based on gold nanoparticles and activated carbon-functionalized chitosan (AC@CS) composite material to evaluate the antioxidant activity of secondary metabolites from endophytic fungi of *Hypericum perforatum* L. Quantum dots, metal nanomaterials, and other nanomaterials with excellent properties, such as high conductivity, biocompatibility, and abundant surface functional groups, are increasingly being applied in the construction of biosensors, which significantly enhances their performance and broadens their applicability.

### Microtechnology and integration

With advancing technology and growing application demands, biosensors are evolving toward greater miniaturization and integration. Miniaturization aims to reduce device size and consumption of samples and reagents, while integration involves the coordinated design of multi-functional modules to achieve portability, automation, and high-throughput operation. For instance, Wei et al. [9] developed a novel target screening method based on virtual screening and fiber-based SPR sensing, which reduces the detection time to 45 min and eliminates the need for large and expensive optical demodulation equipment. The entire device has a compact volume of 5.50 dm^3^. The same research team [120] also designed an all-fiber SPR microfluidic chip for detecting arctigenin, with a fiber diameter of 125 μm. This device is not only small in size but also offers high flexibility, low external interference, and real-time monitoring capabilities. Similarly, Carles et al. [121] developed a silicon-based integrated biosensor system featuring a polymerase chain reaction (PCR) microreactor with an 8 µL reaction chamber and a DNA microarray with 90 spotting sites (each 150 µm in diameter) for DNA-based authentication of toxic Chinese medicinal materials. This system successfully integrated DNA amplification and hybridization analysis on a single microchip, demonstrating the feasibility of miniaturized and functionally integrated biosensing. By combining PCR with microarray detection in a miniaturized device, it embodies the “lab-on—a-chip” concept, providing a technological foundation for rapid and high-throughput identification of herbal materials and showing great potential for field-deployable and portable bioanalysis applications.

The synergistic development of miniaturization and integration is not only advancing TCM research from macro to micro scales but also enabling real-time, in-situ, and dynamic monitoring. However, challenges remain in terms of technical standardization, stability, and cost efficiency, which require further breakthroughs in the future. Overall, the ongoing miniaturization and integration of biosensors are gradually becoming essential technological supports for modernizing TCM research.

### Intelligent data processing

Intelligent data processing technologies have significantly advanced the development of biosensors [122]. At the software level, ML frameworks facilitate intelligent data analysis through a structured pipeline encompassing data preprocessing, feature extraction, and classification modeling. Data preprocessing focuses on improving data quality through procedures such as denoising and normalization. Feature extraction employs methods including time–frequency analysis and principal component analysis (PCA) to identify key patterns within complex datasets.

Classification modeling is subsequently performed using approaches such as deep learning and support vector machines (SVM) to achieve accurate data interpretation and decision-making. Cui et al. [38] developed a SERS biosensor for trace-level detection of amygdalin in TCM and incorporated ML for intelligent data analysis. They applied PCA to reduce the dimensionality of spectral data and used a SVM to classify Raman spectra of Rhodamine 6G at different concentrations, achieving an accuracy of 83%. Subsequently, the same research team [123] employed a similar approach to detect bioactive compounds from TCM, specifically diosgenin and tectorigenin. They further introduced ML methods to process and classify the collected spectral data intelligently. Dimensionality reduction via PCA yielded cumulative contributions of 98% from the first two principal components, effectively extracting spectral features. Among the four classification models evaluated, including decision tree (DT), SVM, naive Bayes, and k-nearest neighbors, the DT model achieved the best performance, with a classification accuracy of 90.91%.These results demonstrate that ML combined with PCA can efficiently process complex SERS spectral data, significantly improving the identification accuracy and reliability of TCM components, and highlight the broad application potential of intelligent algorithms in SERS analysis. Rao et al. [28] integrated ML techniques such as least squares support vector machine (LSSVM) and artificial neural network (ANN) with an electrochemical sensor to construct an intelligent analytical platform. Using Shuanghuanglian oral liquid as a real sample, they verified that the sensor based on an a-MoSₓ-BM modified electrode exhibited high sensitivity and stability in detecting baicalin. Coupled with an LSSVM model, the platform achieved intelligent prediction of concentration from current signals. The results indicated that LSSVM outperformed ANN and traditional linear regression models in predictive accuracy, robustness, and resistance to overfitting, further underscoring the value of ML in enhancing the intelligent analytical capabilities of biosensors.

This innovative AI-integrated paradigm not only significantly improves the detection performance and reliability of biosensors but also enables efficient and accurate extraction of valuable information from complex chemical data, driving the technology toward automation and high precision.

## Summary and outlook

### Limitations and countermeasures of biosensors in TCM research

#### Stability and reproducibility problems

In practical applications, biosensors continue to face two interrelated technical challenges, namely stability and reproducibility. As the core components of biosensors, biorecognition elements, including enzymes, antibodies, and nucleic acids, are highly sensitive to environmental conditions. Factors such as temperature fluctuations, pH variations, and complex sample matrices can significantly compromise their long-term stability and recognition reliability. Temperature, for instance, influences different recognition elements in distinct ways, primarily affecting recognition activity, conformational stability, and binding efficiency. Current strategies for mitigating temperature interference mainly involve structural self-compensation at the sensor level and algorithm-based correction methods. Wei et al. [124] fabricated a temperature-compensated fiber-optic SPR microfluidic sensor using micro- and nanoscale three-dimensional printing technology. In this design, dual detection zones were constructed on opposite sides of a single sensing fiber, and wavelength-division multiplexing was employed to simultaneously acquire optical signals from both regions. This configuration enabled the selective detection of berberine while concurrently monitoring the ambient temperature within the microfluidic channel. By establishing a temperature-compensation matrix, cross-sensitivity caused by temperature variations during the detection process was effectively eliminated. Experimental results demonstrated that the sensor exhibited a temperature sensitivity of 2.18 nm/°C, a detection sensitivity for berberine of 0.2646 μg/mL, and a detection limit as low as 0.38 μg/mL, while maintaining good specificity in mixed solutions.

In addition to temperature effects, the complex composition of TCM sample matrices, including pigments, organic acids, and polysaccharides, poses significant challenges to sensor stability due to nonspecific adsorption, with polysaccharides particularly prone to encapsulating the electrode interface, interfering with recognition element activity, and causing signal drift. Strategies to mitigate these effects can be broadly divided into surface coating modification and sample pretreatment. Antifouling coatings, such as polyethylene glycol (PEG) and hydrophilic polymer brushes, amphoteric materials, and nanostructured surfaces, have been shown to reduce nonspecific adsorption effectively [110, 111], and recent materials advances have further improved their performance. Amphiphilic polymer brushes inhibit nonspecific adsorption primarily through dense hydration layers that sterically hinder protein binding while maintaining electrochemical activity, making them suitable for dilute extracts with low viscosity and limited polyphenol content. Amphoteric materials rely on electrostatic interactions to form stable hydration layers, which are advantageous for high-protein, high-salt matrices, although they are sensitive to extreme pH, high polyphenol concentrations, and relatively costly. Nanostructured surfaces function mainly via physical size exclusion, benefiting high-viscosity decoctions containing large particles, yet their effectiveness against small-molecule interferents is limited, and they are prone to pore blockage and batch-to-batch variability.

Complementing these surface strategies, sample pretreatment reduces matrix interference without altering the sensor surface by optimizing extraction and handling procedures. For example, Li et al. [125] developed an immunoassay-based sensor for rapid detection of matrine in honey, using a monoclonal antibody as the recognition element and gold nanoparticle labeling for signal amplification. Through computer-aided hapten design, highly sensitive and specific antibodies were obtained. The high viscosity and complex matrix of honey were addressed via 50% methanol extraction, ultrasound-assisted release, and five-fold dilution with phosphate-buffered saline (PBS), while surfactants and blocking agents in the resuspension buffer minimized nonspecific adsorption. The sensor achieved detection within 8 min with a visual limit of 2 μg/kg, demonstrating that rational pretreatment can control nonspecific adsorption even without complex coatings, making it suitable for rapid on-site screening. In practice, no single strategy is universally optimal: high-viscosity, polysaccharide-rich samples benefit from nanostructured surfaces combined with sufficient dilution and pretreatment such as sonication or centrifugation; high-protein matrices are better addressed with amphoteric coatings; and acidic, polyphenol-rich extracts are more stably analyzed with amphoteric materials than PEG. Effective analysis generally requires integrating surface modification with appropriate pretreatment, including dilution, surfactants, and blocking agents, to mitigate interference from complex TCM matrices.

Reproducibility constitutes another critical challenge in biosensor applications. Batch-to-batch variations during fabrication, including uneven electrode modification layers, non-uniform dispersion of nanomaterials, and insufficient operational standardization, often lead to deviations in detection results when sensors from the same batch are used to analyze identical samples, thereby compromising quantitative accuracy [126]. Recent advances in material design and fabrication processes have provided promising solutions to improve sensor reproducibility. For example, Wang et al. [127] reported a molecularly imprinted electrochemical sensor based on a biomass-derived carbon platform for detecting glyphosate residues in TCM samples. The sensor exhibited a repeatability RSD of 3.35% and a six-cycle reproducibility RSD of 5.0%, while retaining more than 90% of its initial response after 10 days, demonstrating excellent fabrication consistency and reusability. These findings indicate that strategies such as molecular imprinting can effectively enhance the analytical stability of sensors in complex TCM systems, thereby supporting their practical application.

In summary, stability and reproducibility challenges span the entire lifecycle of biosensor development, from interface construction to practical implementation. Considerable progress has been achieved in addressing temperature fluctuations and nonspecific adsorption, particularly with the rapid development of antifouling interface materials that effectively suppress interferents such as pigments and peptides in complex matrices. Nevertheless, these challenges are often interrelated, and single strategies are typically insufficient to meet the diverse demands of practical sensing applications. Future research should adopt a coordinated approach encompassing three key dimensions: material design, process optimization, and data processing. At the material level, developing novel recognition elements with both antifouling capability and long-term stability remains essential. At the process level, automated manufacturing technologies can reduce batch-to-batch variations and improve reproducibility. At the data-processing level, algorithm-based dynamic correction of signal drift can compensate for hardware limitations. In addition, for high-viscosity samples such as TCM extracts, further optimization of microfluidic interfaces is required to enable stable sample injection and continuous online detection. As these technical bottlenecks are progressively overcome, biosensors are expected to move beyond laboratory proof-of-concept studies toward broader practical applications in complex TCM systems.

#### Barriers to standardization and industrialization

A substantial gap persists between laboratory-level biosensor research and large-scale commercial implementation, particularly regarding standardization and industrial translation. Standardization is essential for ensuring consistency, reliability, and comparability of analytical results across different batches, sources, and time points. Unlike conventional chromatographic techniques, which benefit from well-established international performance criteria, biosensors still lack a unified evaluation framework and standardized operating procedures suitable for complex sample matrices. This limitation stems from variability in the activity, stability, and immobilization efficiency of biological recognition elements, as well as the absence of universally accepted calibration standards or certified reference materials. As a result, data comparability across platforms is limited, which hinders recognition by pharmacopoeias and regulatory authorities.

Industrial translation faces additional challenges. Laboratory fabrication often relies on costly materials and labor-intensive surface modification steps, resulting in high production costs and limited scalability. Achieving ultra-high sensitivity typically requires tightly controlled fabrication processes that are difficult to replicate during scale-up. Moreover, the intrinsic instability of biological recognition elements imposes stringent requirements on storage and shelf life, further complicating commercialization. A more fundamental limitation lies in the mismatch between detection throughput and automation level. Most laboratory-developed biosensors depend on manual operation and sequential detection, where multiple concentration gradients or experimental conditions often require separate runs. In contrast, commercial platforms, such as surface plasmon resonance systems and microfluidic devices, support multi-channel parallel detection, automated sampling, and real-time data acquisition, highlighting the efficiency gap in high-throughput analysis of complex TCM systems.

Several technical issues further limit broader application. Calibration strategies become increasingly complex in multi-analyte TCM extracts, as signal interference undermines conventional single-target approaches. Multivariate calibration or ML-assisted matrix correction methods are therefore necessary. Systematic evaluation of cross-reactivity is also insufficiently standardized; parameters such as selectivity coefficients, cross-reactivity rates, and matrices should be assessed, alongside validation with single interferents, mixed interferents, and spiked recovery experiments in real samples. For high-throughput array-based systems, orthogonal detection channels and competitive inhibition assays are important to confirm signal specificity. Additionally, an inherent trade-off exists between throughput and implementation cost: higher throughput improves sample processing capacity but increases equipment costs, reagent consumption, and data processing demands. Throughput selection should thus be guided by intended applications, ranging from rapid on-site testing to high-precision laboratory analysis.

To overcome these bottlenecks, future research should focus on several key directions: enhancing training programs for specialized biosensor personnel; advancing system-level manufacturing processes and supporting equipment; developing standardized methodological frameworks and performance validation protocols, including specifications for immobilizing biorecognition elements and long-term system stability assessment; promoting integrated sensor development aligned with application needs, combining physical and chemical sensing technologies, and designing multi-parameter analytical instruments; developing online detection technologies to improve production consistency and quality control; and exploring regulatory pathways to facilitate inclusion of mature biosensor technologies in pharmacopoeia appendices or industrial standards. Leveraging the multidisciplinary nature of the field, fostering cross-disciplinary collaboration, and integrating instrument development, application scenarios, and system engineering will be essential to accelerate translation. Ultimately, establishing a comprehensive engineering and quality control framework will drive biosensors toward automated, intelligent, and fully integrated products capable of high-throughput, reliable, and standardized analysis of complex TCM systems.

#### Challenges of AI interpretability

Artificial intelligence (AI) technologies have emerged as powerful tools for modern TCM research by assisting biosensors in elucidating multi-component, multi-target mechanisms through the analysis of high-dimensional datasets and identification of nonlinear patterns (Sect. 4.3). ML algorithms can automatically compensate for sensor drift and environmental fluctuations, converting complex electronic signals into interpretable results for end users. However, these algorithms require prior model training using extensive datasets, with iterative optimization of internal parameters to enhance robustness. Consequently, the quality, consistency, and representativeness of training data are critical. In practical applications, variations in biosensor types, biorecognition elements, and sampling times often yield heterogeneous and limited datasets, substantially reducing algorithm accuracy and adversely affecting prediction and classification performance. Moreover, the intrinsic “black-box” nature of most AI models limits interpretability, posing challenges for mechanistic understanding and experimental validation.

Improving AI interpretability in TCM biosensing requires coordinated strategies across model selection, interpretability tools, and knowledge integration. Inherently interpretable algorithms such as decision trees and logistic regression allow direct examination of the relationship between input features and outcomes, but often fail to capture complex nonlinear patterns, for instance, when distinguishing structurally similar bioactive compounds. Advanced models, including random forests, extreme gradient boosting, and attention-based neural networks, offer higher predictive accuracy but lack explicit decision rules, necessitating complementary interpretation methods. Tools such as Shapley Additive Explanations quantify the contribution of each input feature to model predictions, while attention mechanisms can provide intuitive insights through weight distributions, though their reliability in reflecting true feature importance remains debated. Combining feature importance analysis, attention mechanisms, and knowledge graph-based frameworks allows model decisions to be compared with established TCM theoretical knowledge, enhancing credibility and guiding subsequent experimental validation.

From a data and experimental perspective, biosensor outputs are often highly heterogeneous due to variations in device configurations, sensing elements, and sampling conditions, while available datasets are typically limited in size. Establishing standardized databases that encompass diverse experimental scenarios and data modalities is a fundamental step toward improving the interpretability of AI models. On this basis, data augmentation and transfer learning can further mitigate the constraints imposed by limited data availability. In addition, implementing an experimental feedback loop that continuously compares model predictions with empirical results and refines parameters based on observed discrepancies enables a closed cycle of prediction, validation, and optimization. This iterative strategy helps bridge the gap between theoretical modeling and practical application, thereby progressively enhancing model robustness. Continued advances along these directions are expected to overcome the interpretability challenges of AI-assisted biosensing in TCM, providing both mechanistic insights and practical guidance for experimental design.

### Prospects and outlook of biosensors in TCM research

Biosensors, as analytical tools that translate complex chemical and biological interactions into measurable signals, such as electrical or optical outputs, offer core advantages through multi-target, multifunctional, and multichannel detection capabilities. These features directly address the limitations of traditional methods in capturing the holistic and functional properties of TCM. For example, biosensor arrays can simultaneously detect multiple biomolecular targets, including disease-related receptors, enzymes, or ion channels, thereby more accurately reflecting the unique multi-target synergistic effects characteristic of TCM formulations. Multifunctional biosensors can concurrently monitor multiple physiological parameters, such as neurotransmitter levels, energy metabolism, pH, and temperature, to evaluate systemic regulatory effects under near-physiological conditions. Their applications span a broad spectrum, including medicinal plant identification, active compound screening, target identification, drug–target interaction analysis, quality control, and safety assessment, collectively enhancing objectivity and scientific standardization in the field.

Ongoing advancements in biosensor miniaturization, portability, intelligent data analysis, multifunctional integration, and novel material development are further driving research into the relationship between TCM chemical constituents and therapeutic effects. Looking forward, the integration of sensor technology with the Internet of Things (IoT) and cloud computing is transforming these analytical tools from standalone detection platforms into distributed monitoring nodes. This transition promises to establish a quality assurance network across the entire TCM supply chain, from cultivation and processing to distribution, storage, and clinical application, enabling end-to-end traceability from field to bedside. The convergence of technologies such as microfluidic chips, organ-on-a-chip systems, and AI provides a technical foundation for constructing an IoT-enabled “smart TCM” ecosystem aligned with the holistic evaluation philosophy of TCM. This evolution represents a paradigm shift in quality control, moving from single-compound analysis toward function-oriented, comprehensive assessment.

Nevertheless, this vision faces considerable practical challenges. Issues such as sensor stability, detection reproducibility, and data standardization remain under intensive investigation and are not yet suitable for widespread implementation. Achieving effective solutions requires deep collaboration across multiple disciplines, including biology, materials science, engineering, and regulatory science. As these technical barriers are gradually overcome, biosensors have the potential not only to advance the internationalization of TCM but also to provide more reliable tools for precision and personalized clinical applications.

## Data Availability

No datasets were generated or analysed during the current study.

## Abbreviations

ACE2: Angiotensin-converting enzyme 2
AFB1: Aflatoxin B1
ANN: Artificial neural network
AI: Artificial intelligence
AgNPs: Silver nanoparticles
AuNPs: Gold nanoparticles
CA II: Carbonic anhydrase II
CHA: Catalytic hairpin assembly
DN: Diabetic nephropathy
DON: Deoxynivalenol
DT: Decision tree
EGCG: Epigallocatechin-3-gallate
FET: Field-effect transistors
GDC: Gandi capsule
HEMT: High electron mobility transistors
hQC: Human glutaminyl cyclase
IL-1β: Interleukin-1β
LOD: Limit of Detection
IoT: Internet of Things
K_D_: Dissociation constant
LC–MS: Liquid chromatography–mass spectrometry
LSSVM: Least squares support vector machine
Lut: Luteolin
MAT: Matrine
MIF: Migration inhibitory factor
ML: Machine learning
OPPs: Organophosphate pesticides
OTA: Ochratoxin A
PCA: Principal component analysis
PCR: Polymerase chain reaction
PU: Puerarin
QS: Quorum sensing
RBD: Receptor-binding domain
RQ: Research Questions
RSD: Relative Standard Deviation
SARS-CoV-2: Severe acute respiratory syndrome coronavirus 2
SERS: Surface-enhanced Raman scattering
SiO₂NPs: Silicon dioxide nanoparticles
SPR: Surface plasmon resonance biosensors
sTNFR-I: Tumor necrosis factor's soluble receptor
SVM: Support vector machine
TCM: Traditional Chinese medicine
TiO₂ NPs: Titanium dioxide nanoparticles
TNF-α: Tumor necrosis factor-α
XOD: Xanthine oxidase
ZnO NPs: Zinc oxide nanoparticles
2DEG: Two-dimensional electron gas
